# Supplementary material for: Multi‐dataset identification of innovative feature genes and molecular mechanisms in keratoconus
Source: J Cell Mol Med. 2024 Sep 19;28(18):e70079. doi: 10.1111/jcmm.70079 (PMC11412914; doi:10.1111/jcmm.70079)
Supplement: Supplementary file 2 — Material S2. [file JCMM-28-e70079-s002.zip › 7.GSEA analysis/4.MYRF/KEGG_FATTY_ACID_METABOLISM.html]

Details for gene set KEGG\_FATTY\_ACID\_METABOLISM[GSEA]

|  || Dataset | MYRF.group.cls#h\_versus\_l |
| Phenotype | group.cls#h\_versus\_l |
| Upregulated in class | h |
| GeneSet | KEGG\_FATTY\_ACID\_METABOLISM |
| Enrichment Score (ES) | 0.1954867 |
| Normalized Enrichment Score (NES) | 0.6590028 |
| Nominal p-value | 0.888668 |
| FDR q-value | 1.0 |
| FWER p-Value | 1.0 |
Table: GSEA Results Summary

  

Fig 1: Enrichment plot: KEGG\_FATTY\_ACID\_METABOLISM      
 Profile of the Running ES Score & Positions of GeneSet Members on the Rank Ordered List

  

| SYMBOL | TITLE | RANK IN GENE LIST | RANK METRIC SCORE | RUNNING ES | CORE ENRICHMENT || 1 | ADH6 | na | 1268 | 0.424 | 0.0450 | Yes |
| 2 | ACADL | na | 1353 | 0.414 | 0.1222 | Yes |
| 3 | ACSL6 | na | 2596 | 0.318 | 0.1476 | Yes |
| 4 | CYP4A11 | na | 4635 | 0.234 | 0.1338 | Yes |
| 5 | ADH1B | na | 5054 | 0.221 | 0.1642 | Yes |
| 6 | ADH4 | na | 5377 | 0.211 | 0.1955 | Yes |
| 7 | ACOX1 | na | 7873 | 0.141 | 0.1507 | No |
| 8 | CPT1A | na | 8327 | 0.131 | 0.1628 | No |
| 9 | ACAA2 | na | 9207 | 0.111 | 0.1589 | No |
| 10 | CPT2 | na | 9670 | 0.102 | 0.1652 | No |
| 11 | ACADM | na | 10268 | 0.091 | 0.1656 | No |
| 12 | ACAA1 | na | 10411 | 0.088 | 0.1785 | No |
| 13 | ACOX3 | na | 10534 | 0.086 | 0.1915 | No |
| 14 | ALDH3A2 | na | 11227 | 0.075 | 0.1859 | No |
| 15 | ACSL1 | na | 11845 | 0.066 | 0.1807 | No |
| 16 | ACAT2 | na | 12097 | 0.062 | 0.1854 | No |
| 17 | HADHB | na | 12548 | 0.056 | 0.1832 | No |
| 18 | ECI1 | na | 12908 | 0.051 | 0.1828 | No |
| 19 | ADH7 | na | 14404 | 0.033 | 0.1461 | No |
| 20 | HADHA | na | 16173 | 0.016 | 0.0982 | No |
| 21 | CPT1C | na | 16818 | 0.010 | 0.0816 | No |
| 22 | ACAT1 | na | 17648 | 0.003 | 0.0582 | No |
| 23 | ECHS1 | na | 17726 | 0.002 | 0.0564 | No |
| 24 | ACADSB | na | 18715 | -0.007 | 0.0293 | No |
| 25 | ACADS | na | 19321 | -0.014 | 0.0146 | No |
| 26 | ALDH7A1 | na | 19743 | -0.019 | 0.0060 | No |
| 27 | ACSL4 | na | 19912 | -0.021 | 0.0052 | No |
| 28 | HADH | na | 20242 | -0.025 | 0.0004 | No |
| 29 | ECI2 | na | 20501 | -0.028 | -0.0017 | No |
| 30 | ACADVL | na | 20963 | -0.033 | -0.0086 | No |
| 31 | EHHADH | na | 23596 | -0.069 | -0.0712 | No |
| 32 | ALDH9A1 | na | 23662 | -0.070 | -0.0596 | No |
| 33 | GCDH | na | 23774 | -0.072 | -0.0489 | No |
| 34 | ALDH1B1 | na | 25124 | -0.095 | -0.0694 | No |
| 35 | ACSL5 | na | 25278 | -0.098 | -0.0549 | No |
| 36 | ACSL3 | na | 25434 | -0.102 | -0.0399 | No |
| 37 | CYP4A22 | na | 26880 | -0.132 | -0.0561 | No |
| 38 | ADH1A | na | 26960 | -0.134 | -0.0325 | No |
| 39 | ADH5 | na | 27321 | -0.143 | -0.0154 | No |
| 40 | CPT1B | na | 27722 | -0.153 | 0.0025 | No |
| 41 | ADH1C | na | 33352 | -0.425 | -0.0780 | No |
| 42 | ALDH2 | na | 34342 | -0.612 | 0.0111 | No |
Table: GSEA details [plain text format]

  

Fig 2: KEGG\_FATTY\_ACID\_METABOLISM      
 Blue-Pink O' Gram in the Space of the Analyzed GeneSet

  

Fig 3: KEGG\_FATTY\_ACID\_METABOLISM: Random ES distribution      
 Gene set null distribution of ES for **KEGG\_FATTY\_ACID\_METABOLISM**
